# Heating- and leaching-free separation of electrodes by liquid metals for regeneration of spent Li-ion batteries

**DOI:** 10.1093/nsr/nwag142

**Published:** 2026-03-06

**Authors:** Mingjin Cui, Zhicheng Tian, Yongqing Gong, Bo Xu, Qingtao Gu, Hanjun Li, Xuyang Zhang, Menghao Yang, Ping He, Shixue Dou, Yu Ding

**Affiliations:** Institute of Energy Materials Science, University of Shanghai for Science and Technology, Shanghai 200093, China; National Laboratory of Solid State Microstructures, Collaborative Innovation Center of Advanced Microstructures, College of Engineering and Applied Sciences, Jiangsu Key Laboratory of Nano Technology, Center of Energy Storage Materials & Technology, Nanjing University, Nanjing 210023, China; National Laboratory of Solid State Microstructures, Collaborative Innovation Center of Advanced Microstructures, College of Engineering and Applied Sciences, Jiangsu Key Laboratory of Nano Technology, Center of Energy Storage Materials & Technology, Nanjing University, Nanjing 210023, China; Shanghai Key Laboratory for R&D and Application of Metallic Functional Materials, Institute of New Energy for Vehicles, School of Materials Science and Engineering, Tongji University, Shanghai 201804, China; Institute of Energy Materials Science, University of Shanghai for Science and Technology, Shanghai 200093, China; Institute of Energy Materials Science, University of Shanghai for Science and Technology, Shanghai 200093, China; National Laboratory of Solid State Microstructures, Collaborative Innovation Center of Advanced Microstructures, College of Engineering and Applied Sciences, Jiangsu Key Laboratory of Nano Technology, Center of Energy Storage Materials & Technology, Nanjing University, Nanjing 210023, China; National Laboratory of Solid State Microstructures, Collaborative Innovation Center of Advanced Microstructures, College of Engineering and Applied Sciences, Jiangsu Key Laboratory of Nano Technology, Center of Energy Storage Materials & Technology, Nanjing University, Nanjing 210023, China; College of Smart Energy, Shanghai Jiao Tong University, Shanghai 200240, China; National Laboratory of Solid State Microstructures, Collaborative Innovation Center of Advanced Microstructures, College of Engineering and Applied Sciences, Jiangsu Key Laboratory of Nano Technology, Center of Energy Storage Materials & Technology, Nanjing University, Nanjing 210023, China; Institute of Energy Materials Science, University of Shanghai for Science and Technology, Shanghai 200093, China; National Laboratory of Solid State Microstructures, Collaborative Innovation Center of Advanced Microstructures, College of Engineering and Applied Sciences, Jiangsu Key Laboratory of Nano Technology, Center of Energy Storage Materials & Technology, Nanjing University, Nanjing 210023, China

**Keywords:** heating-free separation, leaching-free separation, regeneration, Li-ion batteries, liquid metals

## Abstract

Efficient separation of aluminum (Al) foil from cathode materials is a key prerequisite for recycling spent Li-ion batteries. Current strategies, relying on thermal treatment or aggressive leaching, suffer from high energy consumption and adverse environmental effects. Here, we present a liquid-metal-induced electrode-separation approach free from heating or leaching. The liquid metal disrupts the passivation layer of the Al and permeates its grain boundaries, enabling the rapid and efficient detachment of active materials. Our combined experimental and computational investigations reveal that the high binding energy between GaSn atoms and the (110) surface of Al drives this grain-boundary diffusion, establishing a foundation for sustainable electrode separation. This approach is universally applicable to widely used cathodes materials, including LiNi_1/3_Co_1/3_Mn_1/3_O_2_ (NCM), LiCoO_2_ (LCO), LiFePO_4_ (LFP) and LiMn_2_O_4_ (LMO). The liquid metal can be instantly regenerated by reacting its dissolved Al with H_2_O, producing high-value H_2_ as a byproduct without harmful emissions. This process achieves a separation efficiency of ∼99.4% for all electrode materials within 30 minutes, maintaining >99.3% efficiency over repeated cycles and demonstrating outstanding reusability. Crucially, the dissolution of transition metals (Ni, Co, Mn, Fe) is negligible, preserving active material integrity. The regenerated NCM, LCO, LFP and LMO cathodes deliver reversible capacities of 172, 148, 144 and 138 mAh g^−1^ at 0.1 C, respectively. Techno-economic assessments corroborate that our liquid-metal-enabled separation surpasses conventional methods, providing a green and cost-effective solution for large-scale battery recycling.

## Teaser

A liquid metal-induced separation strategy enables the rapid, efficient detachment of active materials from aluminum foil, providing a sustainable and universal platform for high-efficiency battery recycling.

## INTRODUCTION

The rapid adoption of electric vehicles and energy-storage solutions has driven a substantial increase in the demand for lithium-ion batteries (LIBs) [1–5]. As these batteries reach the end of their life cycle, their disposal and recycling have emerged as critical industrial concerns [6–9]. Projections indicate that, by 2030, the global volume of discarded LIBs could reach ≥2 million tons annually [10–12], underscoring the urgent need for effective recycling strategies that address both resource sustainability and environmental impact [13,14]. Recycling efforts primarily focus on extracting key components from spent LIBs, particularly cathode materials enriched with valuable metals such as lithium, nickel and cobalt [15–19]. Currently, the most widely employed techniques are hydrometallurgy (wet processing) [20,21] and pyrometallurgy (thermal processing) [22]. While hydrometallurgical methods offer high recovery rates and metal selectivity, they are hindered by significant wastewater generation and complex process control. Conversely, pyrometallurgical processes utilize high-temperature smelting; though straightforward, these methods require intensive energy input. Given that cathode materials account for ≤50% of LIB costs and involve increasingly diverse chemistries, the primary bottleneck in battery recycling remains the efficient separation of active materials from the aluminum (Al) foil current collector [23]. The efficient and sustainable separation of cathode active materials, such as LiNi_1/3_Co_1/3_Mn_1/3_O_2_ (NCM), LiCoO_2_ (LCO), LiFePO_4_ (LFP) and LiMn_2_O_4_ (LMO) from Al foil plays a pivotal role in determining the feasibility and cost-effectiveness of recycling [24,25].

The separation of LIB cathode materials from Al foil can be achieved by using various methods, each with distinct advantages and limitations. Mechanical peeling involves scraping or grinding to detach cathode materials from the Al substrate [26,27]. This method is straightforward and eco-friendly, and preserves the physical properties and morphology of the cathode material; however, it suffers from low efficiency, particularly for strongly adhered electrodes, and is challenging to scale up. Thermal decomposition relies on heating the electrode to degrade or deactivate binders such as polyvinylidene fluoride (PVDF) [28,29], enabling material separation. While scalable and effective for strongly bonded electrodes, this approach risks damaging the active material structure at high temperatures and emitting hazardous gases. Chemical leaching typically employs organic solvents (e.g. *N*-methyl-2-pyrrolidone) or alkaline solutions [30,31] to dissolve the binder. While effective, traditional solvents such as *N*-methyl-2-pyrrolidone face scrutiny due to high costs and toxicity. To mitigate these environmental concerns, recent research has pivoted toward emerging sustainable separation technologies, including deep eutectic solvents (DESs), ionic liquids (ILs) and other green solvents [32–34]. These solvent-chemistry-based delamination methods significantly reduce toxicity and volatility compared with those using *N*-methyl-2-pyrrolidone. However, they fundamentally rely on the chemical dissolution or strong swelling of the PVDF binder to detach the coating. Consequently, these processes are often diffusion-limited and kinetically slow, typically requiring extended soaking times (approximately hours) or elevated temperatures (e.g. 16°C–180°C for DES systems) to overcome the viscosity and transport barriers [33]. Furthermore, the recycling of these complex solvent systems often necessitates energy-intensive purification steps. Electrochemical dissolution alters the electrode potential to dissolve or passivate the Al foil [35]. This method is environmentally friendly and selective, and circumvents high-temperature processing or organic solvents, yet may induce inevitable electrode degradation due to electrochemical oxidation. Consequently, it is eagerly anticipated to develop an innovative and efficient method that is not only scalable and environmentally benign, but also minimizes energy consumption for the separation of active materials and Al foil.

**Figure 1.**
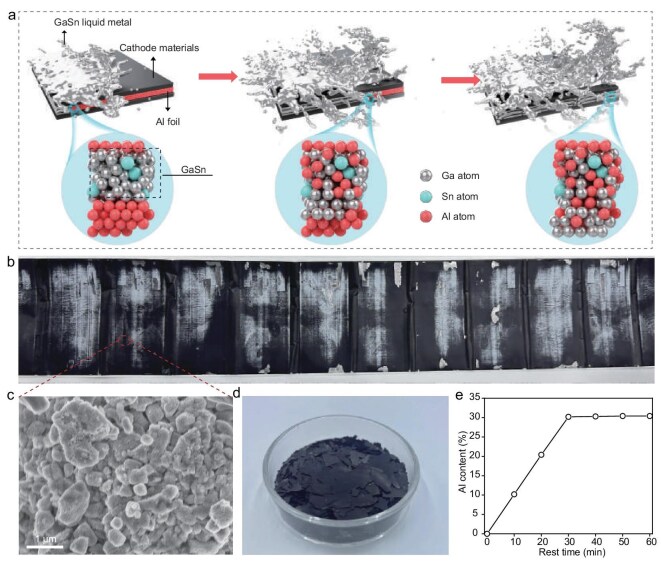
Schematic of liquid-metal-driven separation of Al foil and cathode materials. (a) Schematic illustration of the separation process between Al foil and cathode materials, highlighting the key steps involved. (b) Photograph of the spent cell before the separation process. (c) Scanning electron microscopy (SEM) characterization of the spent electrode materials. (d) Image showing the collected spent LiN_i1/3_Co_1/3_Mn_1/3_O_2_ (NCM) active materials after separation. (e) Plot showing the Al content in the liquid alloy as a function of time, demonstrating the progressive dissolution and incorporation of Al into liquid metals.

Herein, we propose a heating- and leaching-free route to separate LIB electrode materials from Al current collectors through a liquid-metal-enabled strategy that avoids energy-intensive thermal treatment and corrosive chemical leaching (Fig. 1a and Fig. S1). In practical recycling streams, the electrode feedstock is typically obtained after mechanical comminution, in which exposed Al edges and interfacial defects provide efficient entry points for liquid-metal infiltration. Upon contact, the GaSn liquid alloy disrupts the native passivation layer of Al and rapidly propagates along grain boundaries, thereby weakening the interfacial adhesion and enabling the clean detachment of active materials from the Al foil. The reaction breaks up the binding interactions between the Al foil and the active material layer, facilitating a rapid and efficient separation. It is noted that this method proceeds effectively at room temperature, preserving the integrity of sensitive cathode materials and circumventing the electrode damage commonly associated with high-temperature treatments or aggressive chemical leaching procedures. Because liquid metals only selectively etch Al instead of active materials, an exceptional separation efficiency of ∼99.4% can be achieved, making it well suited for processing large-area electrodes. Remarkably, liquid metals can be instantly regenerated via reacting the dissolved Al with H_2_O to remove dissolved Al, and the separation efficiency of recycled liquid metals remains as high as 99.3% over repeated cycles. Herein, the demonstrated outstanding durability and reusability of liquid metals can significantly reduce material expenses for widespread use. Meanwhile, the spontaneous reaction between Al dissolved in liquid metals and water can produce high-value byproducts of hydrogen (H_2_) on demand. Therefore, the liquid-metal-enabled separation process, generating H_2_ without any harmful emissions, adheres to green chemistry principles and supports a sustainable battery-recycling ecosystem. This innovative heating- and leaching-free strategy thus offers a scalable, energy-efficient and environmentally friendly solution for recovering and reusing valuable materials in next-generation battery technologies.

## RESULTS

To assess the feasibility of this technique, we performed separation experiments by using cathodes sourced from retired pouch cells (Fig. 1b). Specifically, when in contact with Al foil, the GaSn liquid alloy rapidly spreads across the Al surface, effectively breaking down the original oxide protective layer. Due to high flowability, Ga and Sn atoms preferentially diffuse along the grain boundaries of Al, causing significant embrittlement. The liquid metal dissolves the aluminum surface, disrupting metallic bonds and reducing the adhesion of the electrode materials to the foil. As a result, the structural integrity of Al is dismantled, leading to the disintegration of the Al foil and further electrode separation. The liquid metal exhibits exceptional separation efficiency, facilitating the thorough and precise removal of cathode materials from the aluminum substrate at scale. The degradation of spent electrode materials brings about irregularly shaped pieces, as evidenced by the digital photograph presented in Fig. 1b and the scanning electron microscopy (SEM) image presented in Fig. 1c, which illustrates the microstructural breakdown of the electrode. Importantly, the separated cathode material, obtained by the liquid-metal-induced approach, exhibits an exceptionally clean surface that is completely free of residual Al, as shown in Fig. 1d. Additionally, energy-dispersive X-ray spectroscopy (EDS) analysis of the detached active materials confirms the absence of Al residues (Figs S2 and S3), underscoring the chemical selectivity of the liquid metal. In contrast, other reported methods inevitably give rise to some Al residues (∼2 wt%) in the active material, complicating the subsequent recycling process [36–38]. Therefore, it is validated that liquid metals can effectively remove Al foil while preserving the integrity of the active materials for further recycling and reuse. Besides, the Al content in liquid metals was monitored during the dissolution process (Fig. 1e), revealing that the Al content could reach ∼30 at%. This progressive dissolution and incorporation of Al into the liquid metals further demonstrates their strong capacity to dissolve Al, a property that can effectively facilitate electrode separation. The liquid-metal-based methodology provides a sustainable solution for recycling battery components. Such advances hold substantial potential for resource recovery and waste minimization, particularly in the rapidly growing market of electric vehicles and stationary energy storage.

**Figure 2.**
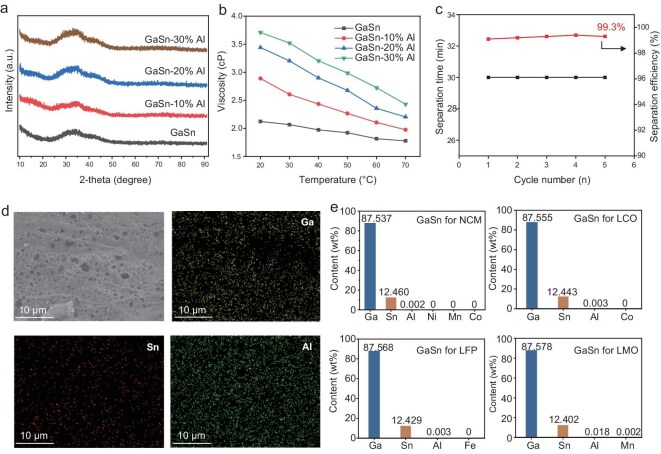
Separation mechanism of Al foil from cathode material using GaSn liquid alloy. (a) X-ray diffraction (XRD) patterns of GaSn liquid alloy with varying Al content. (b) Viscosity of GaSn with different Al contents as a function of temperature. (c) Separation efficiency vs. cycle number for separation of Al foil from the cathode material layer using GaSn liquid alloy. (d) SEM image of GaSn with 30% Al and EDS mapping of Ga, Sn and Al elements. (e) Inductively coupled plasma mass spectrometry characterization of GaSn–Al and the elemental content in electrode materials (NCM, LCO, LFP, LMO) after separating electrode materials and Al using GaSn, followed by reaction with water.

To probe into the structural evolution of liquid metals during the cathode materials separation process, X-ray diffraction (XRD) patterns were collected for GaSn liquid alloys incorporating different Al contents. As shown in Fig. 2a, the liquid alloy consistently exhibits an amorphous structure, irrespective of the Al content. This structural feature suggests that the alloy remains disordered and Al is incorporated into the GaSn matrix to form a homogeneous and metastable amorphous phase or ultra-fine nanoclusters, rather than forming intermetallic compounds or simple floating particles [39]. This atomic-level interaction balances viscosity and flowability (Fig. 2b), maintaining the liquid state at operating temperatures, as confirmed by differential scanning calorimetry tests (Fig. S4). Based on these structural insights, we propose a separation mechanism governed by the dissolution of Al in liquid metals and the resulting weakened binding force between Al and the cathode coating. The process is initiated by surface depassivation, in which the GaSn liquid alloy rapidly wets the Al surface to disrupt the native oxide layer. Subsequently, Al dissolved in GaSn enables the liquid metal to preferentially diffuse along the Al grain boundaries—a behavior driven by the strong thermodynamic affinity between GaSn and Al. This leads to liquid-metal embrittlement in which the intergranular cohesion is drastically reduced. Finally, the Al foil undergoes structural disintegration and amorphization as the detached grains are enveloped by the liquid metals. This mechanism enables the alloy to infiltrate the electrode surface effectively, playing a key role in achieving efficient and rapid separation, as evidenced by the effective detachment of the cathode material from the Al foil within a 30 minutes timeframe (Fig. 2c). These results highlight the effectiveness of the proposed method in electrode separation. Meanwhile, the separation efficiency remains at ∼99.3% over multiple cycles, demonstrating the robustness and repeatability of the approach. Such efficiency is attributed to the ability of liquid metals to rapidly dismantle Al foil via grain-boundary infiltration without damaging the underlying active materials, manifested by the clean morphology of the separated electrode materials (Figs S2 and S3). SEM and EDS mapping of the liquid metal incorporating Al (Fig. 2d) further confirms the clean separation process, with the elemental maps showing a uniform distribution of Ga, Sn and Al atoms. Furthermore, EDS analysis (Figs S5 and S6) shows a corresponding increase in Al concentration with the rising Al content. Even at the high ratio of 30%, the Al species remain evenly dispersed in the sea of Ga and Sn, suggesting the high efficacy of liquid metals in separating electrodes.

It is noted that the liquid metals containing dissolved or amorphous Al can be regenerated after reacting with water, producing high-value products of H_2_ and refreshed liquid metals for continuous electrode separation. To evaluate the separation efficiency and recyclability of the liquid-metal-enabled heating- and leaching-free cathode separation process, inductively coupled plasma mass spectrometry (ICP–MS) was employed. Figure 2e provides the composition of the regenerated liquid metals after separating different cathodes, namely NCM, LCO, LFP and LMO. Impressively, the liquid-metal content remains almost constant before (Fig. S7) and after regeneration (Fig. 2e) with nearly undetectable Al content, indicating the remarkable recyclability of liquid metals. This finding highlights the potential of this approach for efficient electrode separation and Al removal, which is critical to sustainable material recovery. Moreover, ICP–MS analysis reveals that the concentrations of transition metals in the regenerated liquid metals, such as Ni, Co, Mn and Fe from cathodes, are almost negligible (all <0.002 wt%). This compelling result suggests that the liquid-metal-induced process can achieve the nondestructive separation of Al foil from the active material layer. Together, these findings illustrate a highly efficient, selective and recyclable approach for separating Al foil from active materials in retired battery electrodes, offering a scalable and environmentally friendly solution for battery recycling.

In addition to high separation efficiency and recyclability, a high-value byproduct of H_2_ can be derived during the regeneration of liquid metals. To monitor and quantify the generated H_2_, we utilized a high-precision gas detector, as shown in the inset of Fig. S8. The detector tracked the dynamic changes in gas production, capturing real-time data that allowed us to observe the progression of the reaction. As depicted in Fig. S8, the gas-production rate exhibited a clear correlation with time, providing valuable insights into the efficiency of the reaction process. This H_2_-generation reaction between Al and water is driven by GaSn liquid metals, which can form micro galvanic cells given the higher reducibility of Al than Ga and Sn [40]. Meanwhile, liquid metals can disrupt the passivation layer on Al and suppress further passivation of its grain surfaces after permeation into Al grain boundaries. The generated amorphous Al species in the sea of Ga and Sn (Fig. 2d) give rise to more reactive sites and surfaces for H_2_ generation. Notably, H_2_ currently holds significant economic value due to its potential as a clean energy carrier, making it valuable for decarbonizing various industries, particularly in transportation, power generation and heavy industry. This H_2_ co-product from the liquid-metal-enabled electrode-separation approach further highlights its great potential for practical applications, maximizing the utilization of resources while maintaining sustainability in the meantime.

### Density functional theory calculations of Al interaction with GaSn liquid alloys

**Figure 3.**
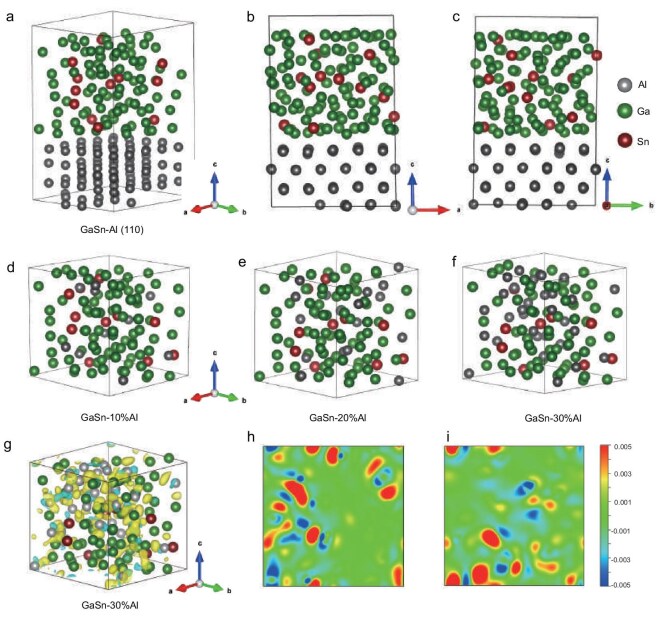
DFT calculations of Al interaction with GaSn liquid alloys. (a) Schematic representation of the Al (110) facet interfaced with the GaSn liquid alloy. (b and c) Side-view snapshots of the structure shown in (a), illustrating the interaction between the Al (110) surface and the GaSn liquid alloy from different angles. (d–f) Structural models of GaSn alloys doped by 10%, 20% and 30% Al. (g) Visualization of the GaSn–Al alloy structure, where contours represent the charge-density distribution. (h and i) Top-view and side-view representations of the charge-density contours in the GaSn–Al alloy.

Density functional theory (DFT) calculations were further conducted to monitor the atomic-level interactions between Al and liquid metals during electrode separation. Based on binding-energy calculations, we found that the GaSn liquid alloy exhibits the lowest binding energy with Al on the (110) surface (–14.93 eV), making it the most favorable surface for Al incorporation compared with the (100) and (111) planes, which have binding energies of –11.77 and –14.66 eV, respectively. Figure 3a presents a schematic diagram of the GaSn–Al (110) interface, along with alternative views (Fig. 3b and c) to highlight the GaSn–Al interaction at this surface. The high binding energy suggests a more intimate GaSn–Al interface in comparison with the Al–Al interface, driving the permeation of the GaSn liquid alloy into the Al grain boundaries and further electrode separation. In addition to surface interactions, we examined the dissolution of Al at varying concentrations (10%, 20% and 30%) within the GaSn liquid alloy (Fig. 3d–f). The results reveal that Al atoms are uniformly dispersed throughout the liquid alloy, indicating a stable and homogeneous distribution that is consistent with the EDS analysis shown in Fig. 2d. The digestion of Al foil modifies liquid metals via the substitution or interstitial occupation of GaSn alloys by Al atoms, which leads to distortions in the electron density and promotes short-range atomic ordering. Further analysis of the charge-density distribution after Al doping shows a clear charge-redistribution process (Fig. 3g–i). Specifically, Al atoms experience a reduction in charge density, indicating electron loss during the doping process. This electron loss is counterbalanced by the gain of electrons by the Ga and Sn atoms close to the Al, leading to an increase in their local charge density. This electron redistribution not only reflects the electron-donating nature of Al, but also influences the electronic structure of the GaSn alloy. The charge distribution reflects enhanced atomic interactions and short-range ordering, which in turn lead to the high Al-incorporation capability of liquid metals. In light of the experimental and computational results, we propose that the electron-donating nature of Al, along with the liquid nature of the GaSn alloy, is key to the efficient infiltration of liquid metals and rapid separation of electrodes. The electron redistribution enables the liquid metal to effectively wet the Al interface, penetrate the grain boundaries and disrupt the metallic bonds. As a result, the Al foil is dismantled and detached from the electrode materials. These findings provide insights into the reaction pathways and transition states of the liquid-metal-induced reactions at the atomic scale and such dynamic evolution reveals how the operational chemical and electronic structure governs the electrode-separation process.

### Electrochemical performance of regenerated cathode materials

**Figure 4.**
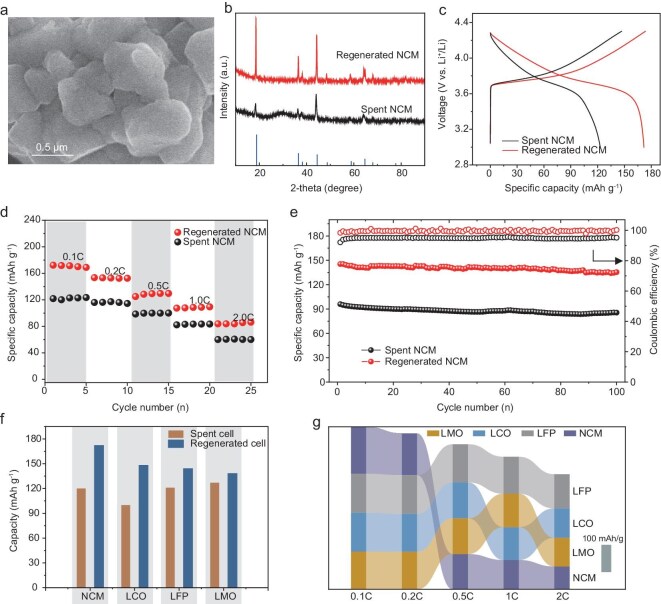
Characterizations of regenerated active material NCM. (a) SEM image and (b) XRD patterns of the regenerated NCM. (c) Voltage-capacity profiles of the regenerated NCM at 0.1 C. (d) Rate performance at various current densities (0.1, 0.2, 0.5, 1.0 and 2.0 C). (e) Comparison of the cycling performance at 0.2 C between the degraded and regenerated NCM. (f) Capacity comparison at 0.1 C of spent cells (NCM, LCO, LFP and LMO) and their regenerated counterparts. (g) Rate performance of the various regenerated cathode materials.

The use of GaSn liquid metals allows the separation of Al foil from spent cathode materials free from heating or leaching and this process preserves the structural integrity and compositional nature of electrode materials. Therefore, the liquid-metal-induced electrode-separation approach creates a solid foundation for the efficient regeneration of cathodes through direct annealing in the presence of lithium salts. Herein, we demonstrate the efficient separation and regeneration of all the widely employed cathode materials in the market, namely NCM, LCO, LFP and LMO. The SEM image shown in Fig. 4a reveals that cracks in spent cathode material (using NCM as an example) particles were effectively repaired during the recovery process, resulting in a dense particle structure. XRD analysis (Fig. 4b) confirms the restoration of a pure layered *α*-NaFeO_2_ phase—evidence of successful material recovery. The electrochemical performance of the regenerates NCM demonstrates significant improvements. As shown in Fig. 4c, the discharge capacity of the regenerates NCM increases remarkably compared with that of the spent electrode. Furthermore, the regenerated NCM exhibits superior rate performance, achieving discharge capacities of 172, 153, 125, 107 and 84 mAh g^−1^ at 0.1, 0.2, 0.5, 1 and 2 C, respectively—far surpassing the capacities of the spent NCM under the same conditions (e.g. 121 mAh g^−1^ at 0.1 C and 60 mAh g^−1^ at 2 C, Fig. 4d). Additionally, the regenerated NCM maintains a high capacity retention of 96.5% over 100 cycles at 0.2 C, as depicted in Fig. 4e. To validate the versatility of the liquid-metal-induced electrode separation–regeneration process, we applied the same regeneration strategy to a range of other cathode materials, including LCO, LFP and LMO (Fig. 4f). Detailed test results for these materials are provided in Fig. 4g and Figs S9–S20. Significant capacity improvements were observed across all materials. Moreover, the regenerated materials demonstrate exceptional cycle stability, as shown in Fig. S21. After 60 cycles, the capacity retention rates were 98.8%, 97.6%, 98.9% and 94.6% for NCM, LCO, LFP and LMO, respectively. Such remarkable regeneration performance should be ascribed to the efficient separation between Al and the electrode materials while preserving the structural and chemical integrity of the cathodes. The liquid-metal-induced separation method can circumvent the possible electrode damage associated with thermal treatment or aggressive chemical leaching, offering a promising pathway for the sustainable recycling of spent batteries.

### Techno-economic analysis

To contextualize our liquid-metal-enabled strategy within the evolving landscape of sustainable recycling, we first qualitatively benchmarked it against emerging solvent-based technologies, including DESs and ILs, as detailed in Supplementary Table S1. While these nascent methods offer reduced toxicity compared with those involving traditional organic solvents, they often incur hidden energetic costs due to the high viscosity of the fluids and the elevated temperatures (typically 160°C–180°C) required to drive binder-dissolution kinetics [41]. In contrast, our approach relies on physical–chemical interfacial displacement rather than chemical dissolution, enabling rapid separation (<30 min) at ambient temperatures. This fundamental mechanistic advantage allows a simplified process flow that avoids energy-intensive solvent heating and recovery steps (Table S2).

**Figure 5.**
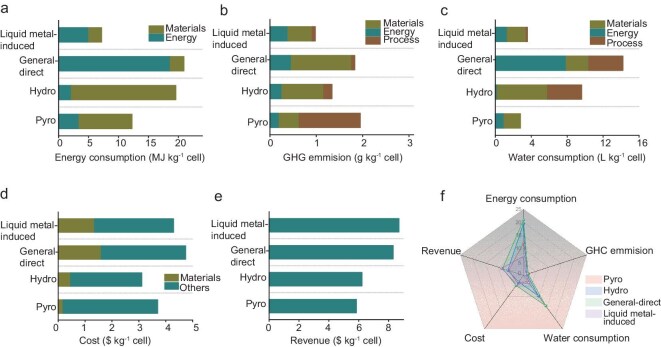
Techno-economic analysis of liquid-metal-induced and other recycling approaches. (a) Energy consumption associated with different recycling methods. (b) Greenhouse gas (GHG) emissions for liquid-metal-induced, Direct, Hydro and Pyro approaches. (c) Water-consumption comparison across various recycling techniques. (d) Cost analysis for each recycling method, highlighting differences in resource utilization and process efficiency. (e) Revenue generated from the recycling of GaSn using the liquid-metal-induced, Direct, Hydro and Pyro methods. (f) Comprehensive comparison of the economic and environmental impacts of different recycling approaches, integrating energy, GHG emissions, water use, cost and revenue for a holistic evaluation.

Building on these process advantages, we conducted a rigorous quantitative techno-economic analysis comparing our method against established industrial baselines: general-direct recycling (Direct), pyrometallurgical recycling (Pyro) and hydrometallurgical recycling (Hydro). As shown in Fig. 5a, the liquid-metal-induced approach demonstrates a significantly lower total energy consumption of 7.18 MJ kg^−1^ cell compared with Direct (20.92 MJ kg^−1^), Hydro (19.57 MJ kg^−1^) and Pyro (12.26 MJ kg^−1^). This energy reduction stems from the unique properties of the GaSn liquid alloy, which facilitates the direct separation of active materials without the need for energy-intensive procedures such as shredding, thermal treatment or smelting. In addition to energy savings, the liquid-metal-induced approach achieves the lowest greenhouse gas (GHG) emissions of 0.98 g kg^−1^ cell (Fig. 5b). This stark contrast versus Direct (1.83 g kg^−1^), Hydro (1.34 g kg^−1^) and Pyro (1.95 g kg^−1^) can be attributed to the reuse of GaSn liquid metals, which eliminates the need for additional combustion processes or pyrometallurgical processes commonly associated with Hydro and Pyro methods. The closed-loop system for the GaSn liquid alloy avoids carbon-intensive steps, making it a more sustainable option for large-scale recycling. The liquid-metal-induced method also demonstrates superior performance in water usage, consuming only 3.57 L kg⁻^1^ cell (Fig. 5c). This is comparable to Pyro (2.80 L kg^−1^) but drastically lower than Hydro (9.61 L kg^−1^) and Direct (14.21 L kg^−1^). This advantage is primarily due to the recyclability of the liquid alloy, which minimizes the reliance on water for washing or chemical processing. Furthermore, the reduced need for secondary separation processes curtails water-intensive operations, aligning the method with sustainable water-management goals. Figure 5d shows that the total cost of the liquid-metal-induced method is $4.29 kg^−1^ cell. As Ga, the main element of liquid metals, is primarily obtained as a byproduct from the extraction of other metals, its market price is largely influenced by the market conditions of the main metals. Although the upfront material cost of the liquid alloy is relatively high at the current stage, its ability to be reused across multiple recycling cycles significantly lowers the long-term cost. Besides, it is noted that Ga is almost as abundant as Cu and Zn in Earth’s crust (Fig. S22), suggesting the potential for a large supply and lower costs in the future [42,43]. In comparison, Direct, Hydro and Pyro incur higher costs of $4.74, $3.11 and $3.70 kg^−1^, respectively, due to higher energy and material demands. Despite its modest cost, the liquid-metal-induced approach provides the highest revenue at $8.70 kg^−1^ cell, as shown in Fig. 5e, outpacing Direct ($8.31), Hydro ($6.22) and Pyro ($5.85). This superior revenue generation is largely attributed to the high purity and excellent performance of the regenerated cathode materials achieved via the liquid-metal-induced approach, as well as the byproduct of high-value H_2_ without any harmful emissions. Meanwhile, unlike traditional methods such as Hydro or Pyro, which often result in the degradation of material properties (e.g. structural damage or loss of electrochemical performance), the liquid-metal-induced method minimizes material degradation during recycling. By utilizing the gentle and selective separation properties of liquid alloy, the process preserves the chemical integrity and crystalline structure of active materials, ensuring high-quality outputs. This capability not only enhances the performance of recycled materials, but also increases their market value, further underscoring the economic advantage of this approach. Figure 5f summarizes these metrics in a radar plot, in which the liquid-metal-induced method exhibits superior performance across all categories, including energy efficiency, GHG emissions, water consumption, cost and revenue. The reusability of liquid metal is a critical enabler, allowing this approach to achieve a closed-loop recycling system with minimal environmental impacts and maximum economic returns. Moreover, its straightforward process design reduces the complexity of operation, making it highly adaptable for industrial-scale battery recycling.

## CONCLUSIONS

The liquid-metal-enabled separation method for Al foil and active cathode materials presents a promising and sustainable solution for the large-scale recycling of LIBs. By circumventing the need for high-temperature treatment or aggressive leaching procedures, this approach significantly reduces energy consumption and minimizes harmful environmental impacts, aligning with green chemistry principles. The method achieves >99.4% separation efficiency with consistent performance over repeated cycles, demonstrating its scalability and eco-friendliness. Moreover, the liquid-metal-based approach ensures negligible dissolution of valuable transition metals such as Ni, Co, Mn and Fe, preserves the structural integrity and compositional nature of cathodes and circumvents the electrode damage commonly associated with thermal treatment or leaching procedures. The approach based on liquid metals represents a universal methodology for recycling all the commonly used cathode materials in the market, and the resulting regenerated electrodes, including NCM, LCO, LFP and LMO, all deliver decent electrochemical performance with high reversible capacities. Notably, liquid metals can be instantly regenerated by reacting with H_2_O to remove the dissolved Al. This reaction produces high-value byproducts of H_2_ without generating any harmful emissions. Techno-economic assessments further highlight the advantages of this method in terms of energy efficiency, environmental impacts and cost-effectiveness compared with conventional recycling techniques. Overall, this liquid-metal-based separation technology offers a highly effective, environmentally benign and economically viable pathway for advancing sustainable battery-recycling practices.

## METHODS

### Separation of Al foil and NCM, LCO, LFP, LMO active material layers using liquid metals

Representative cells from each batch were fully discharged to 0 V for safety considerations and then disassembled to recover the cathode materials, including NCM, LCO, LFP and LMO. The retrieved electrodes were rinsed with ethanol and dried overnight at 60°C prior to further processing. The reaction of the spent electrodes with GaSn liquid metal and the subsequent regeneration of the liquid metal proceeded as follows. (i) Initial liquid metal: pristine GaSn alloy was prepared as the reactive medium. (ii) Immersion and reaction: the dried cathode materials were completely immersed in the GaSn liquid metal and subjected to mechanical ultrasonication for 30 minutes, facilitating intimate interfacial contact and efficient dissolution of the Al foil current collector. The intact spent electrode sheet could be mechanically shredded before the immersion reaction. (iii) Post-reaction state: upon reaction, the liquid metal exhibited minor compositional and color variations owing to aluminum incorporation. (iv) Mesh filtration: the resultant mixture was separated by using filtration, ensuring the complete separation of the cathode powders from the reacted liquid alloy. (v) Collection and drying: the separated cathode materials were repeatedly washed with ethanol and dried at 60°C to yield purified electrode materials. (vi) Regeneration of liquid metal: the spent GaSn alloy was regenerated via a reaction with deionized water, during which the dissolved Al rapidly reacted to form Al_2_O_3_ and H_2_ gas. The Al_2_O_3_ byproduct was removed by using centrifugation, restoring the GaSn liquid metal to its pristine state. The regenerated GaSn liquid metal was directly reused for subsequent recovery cycles.

### Determination of separation efficiency

We measured the mass of the cathode electrode sheet before separation (*m*_total_) and the mass of the recovered cathode coating layer after separation and drying (*m*_recovered_). The efficiency (*η*) is defined as:


\begin{eqnarray*}
\eta = \left( {{m}_{{\mathrm{recovered}}}/{m}_{{\mathrm{coating}}}} \right) \times 100\%.
\end{eqnarray*}

The *m*_coating_ value was determined by subtracting the theoretical mass of the Al foil from the *m*_total_ value.

Detailed methods are available in the online Supplementary file.

## Supplementary Material

nwag142_Supplemental_File

## References

[1] Gibb BC . The rise and rise of lithium. Nat Chem 2021; 13: 107–9. 10.1038/s41557-021-00638-w 33514935

[2] Kim J-H, Kim N-Y, Ju Z et al. Upscaling high-areal-capacity battery electrodes. Nat Energy 2025; 10: 295–307. 10.1038/s41560-025-01720-0

[3] Qi F, Li Q, Zhang W et al. Freestanding ReS_2_/graphene heterostructures as binder-free anodes for lithium-ion batteries. ACS Appl Mater Interfaces 2023; 15: 21162–70. 10.1021/acsami.3c02321 37079857

[4] Sun JY, Li YC, Lv LZ et al. Selective adsorption of electrolyte anions with chitosan skin producing LiF-enriched solid electrolyte interphase for Si-based lithium-ion batteries. Adv Funct Mater 2024; 34: 2410693. 10.1002/adfm.202410693

[5] Wang T, Chen B, Liu Y et al. Fatigue of Li metal anode in solid-state batteries. Science 2025; 388: 311–6. 10.1126/science.adq6807 40245125

[6] Luo P, Su KX, Wu L et al. Solid polymer electrolyte with dual Lewis-acid filler for ultra-stable lithium metal batteries. Adv Mater 2025; 37: 2501142. 10.1002/adma.202501142 40285381

[7] Li DY, Yu DF, Zhang GW et al. High configuration entropy promises electrochemical stability of chloride electrolytes for high-energy, long-life all-solid-state batteries. Angew Chem Int Ed 2025; 64: e202419735. 10.1002/anie.202419735 39431985

[8] Tan S-J, Wang W-P, Tian Y-F et al. Advanced electrolytes enabling safe and stable rechargeable Li-metal batteries: progress and prospects. Adv Funct Mater 2021; 31: 2105253. 10.1002/adfm.202105253

[9] Li XF, Shi XY, Lee SY et al. Closing the loop: universal regeneration and upcycling of spent lithium-ion battery cathodes. Natl Sci Rev 2025; 12: nwaf445. 10.1093/nsr/nwaf445 41268197 PMC12628746

[10] Wang J, Ma J, Zhuang Z et al. Toward direct regeneration of spent lithium-ion batteries: a next-generation recycling method. Chem Rev 2024; 124: 2839–87. 10.1021/acs.chemrev.3c00884 38427022

[11] Liu KL, Shang YL, Ouyang Q et al. A data-driven approach with uncertainty quantification for predicting future capacities and remaining useful life of lithium-ion battery. IEEE Trans Ind Electron 2021; 68: 3170–80. 10.1109/TIE.2020.2973876

[12] Zhang H, Ji Y, Yao Y et al. Transient and dry recycling of battery materials with negligible carbon footprint and roll-to-roll scalability. Energy Environ Sci 2023; 16: 2561–71. 10.1039/D2EE03910A

[13] Zheng MT, You Y, Lu J. Understanding materials failure mechanisms for the optimization of lithium-ion battery recycling. Nat Rev Mater 2025; 10: 355–68. 10.1038/s41578-025-00783-5

[14] Lai ZY, Long J, Lu Y et al. Direct recycling of retired lithium-ion batteries: emerging methods for sustainable reuse. Adv Energy Mater 2025; 15: 2501009. 10.1002/aenm.202501009

[15] Wang JX, Ji HC, Li JF et al. Direct recycling of spent cathode material at ambient conditions via spontaneous lithiation. Nat Sustain 2024; 7: 1283–93. 10.1038/s41893-024-01412-9

[16] Yin YC, Li C, Hu XS et al. Rapid, direct regeneration of spent LiCoO_2_ cathodes for Li-ion batteries. ACS Energy Lett 2023; 8: 3005–12. 10.1021/acsenergylett.3c00635

[17] Fan ES, Li L, Wang ZP et al. Sustainable recycling technology for Li-ion batteries and beyond: challenges and future prospects. Chem Rev 2020; 120: 7020–63. 10.1021/acs.chemrev.9b00535 31990183

[18] Guo YQ, Yao YG, Guo C et al. Atomistic observation and transient reordering of antisite Li/Fe defects toward sustainable LiFePO_4_. Energy Environ Sci 2024; 17: 7749–61. 10.1039/D4EE01622J

[19] Wang XT, Gu ZY, Cao JM et al. Dual-loop upcycling of spent LiFePO_4_: defect inheritance enables durable and fast-charging sodium-ion batteries. Natl Sci Rev 2025; 12: nwaf321. 10.1093/nsr/nwaf321 40937444 PMC12421578

[20] Zhang S, Gu K, Ba L et al. Hydrometallurgical processes on recycling of spent lithium-ion battery cathode: advances and applications in sustainable technologies. Acta Phys Chim Sin 2024; 40: 2309028. 10.3866/PKU.WHXB202309028

[21] Sattar R, Ilyas S, Bhatti HN et al. Resource recovery of critically-rare metals by hydrometallurgical recycling of spent lithium ion batteries. Sep Purif Technol 2019; 209: 725–33. 10.1016/j.seppur.2018.09.019

[22] Pan C, Shen YF. Pyrometallurgical recycling of spent lithium-ion batteries from conventional roasting to synergistic pyrolysis with organic wastes. J Energy Chem 2023; 85: 547–61. 10.1016/j.jechem.2023.06.040

[23] Fang ZW, Zhu P, Zhang X et al. Self-looped electrochemical recycling of lithium-ion battery cathode materials to manufacturing feedstocks. Nat Chem Eng 2025; 2: 142–51. 10.1038/s44286-025-00186-x

[24] Chen Z, Feng R, Wang W et al. Reaction-passivation mechanism driven materials separation for recycling of spent lithium-ion batteries. Nat Commun 2023; 14: 4648. 10.1038/s41467-023-40369-9 37532688 PMC10397256

[25] Yang F, Chen X, Qu G et al. Electrode separation via water electrolysis for sustainable battery recycling. Nat Sustain 2025; 8: 520–9. 10.1038/s41893-025-01539-3

[26] Rubens GZ, Noel L, Sovacool BK. Dismissive and deceptive car dealerships create barriers to electric vehicle adoption at the point of sale. Nat Energy 2018; 3: 501–7. 10.1038/s41560-018-0152-x

[27] Fan E, Li L, Zhang X et al. Selective recovery of Li and Fe from spent lithium-ion batteries by an environmentally friendly mechanochemical approach. ACS Sustain Chem Eng 2018; 6: 11029–35. 10.1021/acssuschemeng.8b02503

[28] Ji H, Wang J, Ma J et al. Fundamentals, status and challenges of direct recycling technologies for lithium ion batteries. Chem Soc Rev 2023; 52: 8194–244. 10.1039/D3CS00254C 37886791

[29] Yang Y, Huang G, Xu S et al. Thermal treatment process for the recovery of valuable metals from spent lithium-ion batteries. Hydrometallurgy 2016; 165: 390–6. 10.1016/j.hydromet.2015.09.025

[30] Yang C, Zhang J, Yu B et al. Recovery of valuable metals from spent LiNi_*x*_Co_*y*_Mn_*z*_O_2_ cathode material via phase transformation and stepwise leaching. Sep Purif Technol 2021; 267: 118609. 10.1016/j.seppur.2021.118609

[31] Fan X, Song C, Lu X et al. Separation and recovery of valuable metals from spent lithium-ion batteries via concentrated sulfuric acid leaching and regeneration of LiNi_1/3_Co_1/3_Mn_1/3_O_2_. J Alloys Compd 2021; 863: 158775. 10.1016/j.jallcom.2021.158775

[32] Cheng MQ, Ru JJ, Miao SK et al. Hydration equilibrium-controlled cation-anion coordination competition for precise recovery of all valuable metals from spent lithium-ion batteries. Energy Environ Sci 2025; 18: 10473–82. 10.1039/D5EE05276A

[33] Hu Y, Yang MC, Dong QY et al. Green and sustainable recycling of lithium-ion batteries via an ionic liquid-driven cathode reduction method. Energy Environ Sci 2024; 17: 4238–47. 10.1039/D4EE00331D

[34] Li PW, Xu HY, Luo SH et al. Green and non-destructive separation of cathode materials from aluminum foil in spent lithium-ion batteries. Sep Purif Technol 2024; 338: 126625. 10.1016/j.seppur.2024.126625

[35] Kwak W, Ha J, Lee J et al. Sustainable recycling of lithium-ion battery cathodes through facile electrochemical delamination. J Power Sources 2024; 617: 235102. 10.1016/j.jpowsour.2024.235102

[36] Zhang HJ, Chen X, Zou X et al. Research progress of aluminum removal technology for cathode materials of spent lithium-ion batteries. J Process Eng 2020; 20: 503–9.

[37] Sun XD, Vitalii I. Environmental impact analysis of waste lithium-ion battery cathode recycling. J Ecol Eng 2024; 25: 352–8.

[38] Zhang BC, Xu YL, Silvester DS et al. Direct regeneration of cathode materials in spent lithium-ion batteries toward closed-loop recycling and sustainability. J Power Sources 2024; 589: 233728. 10.1016/j.jpowsour.2023.233728

[39] Amberchan G, Lopez I, Ehlke B et al. Aluminum nanoparticles from a Ga-Al composite for water splitting and hydrogen generation. ACS Appl Nano Mater 2022; 5: 2636–43. 10.1021/acsanm.1c04331

[40] Zhang X, Fang J, Feng Y et al. The mechanism of water decomposition on surface of aluminum and gallium alloy during the hydrogen production process: a DFT study. Int J Hydrogen Energy 2024; 66: 354–61. 10.1016/j.ijhydene.2024.04.107

[41] Wang JX, Lyu Y, Zeng R et al. Green recycling of spent Li-ion battery cathodes via deep-eutectic solvents. Energy Environ Sci 2024; 17: 867–84. 10.1039/D3EE02978F

[42] Ding Y, Guo XL, Yu GH. Next-generation liquid metal batteries based on the chemistry of fusible alloys. ACS Cent Sci 2020; 6: 1355–66. 10.1021/acscentsci.0c00749 32875076 PMC7453561

[43] Ding Y, Guo XL, Qian YM et al. Room-temperature all-liquid-metal batteries based on fusible alloys with regulated interfacial chemistry and wetting. Adv Mater 2020; 32: 2002577. 10.1002/adma.202002577 32548922

