# Supplementary material for: Heating- and leaching-free separation of electrodes by liquid metals for regeneration of spent Li-ion batteries
Source: Natl Sci Rev. 2026 Mar 6;13(9):nwag142. doi: 10.1093/nsr/nwag142 (PMC13188993; doi:10.1093/nsr/nwag142)
Supplement: nwag142_Supplemental_File [file nwag142_supplemental_file.pdf]

Supplementary Information for

Heating- and leaching-free separation of electrodes by liquid metals for regeneration of spent Li-ion batteries

Mingjin Cui,[1,2,†] Zhicheng Tian,[2,†] Yongqing Gong,[3,†] Bo Xu,[1,†] Qingtao Gu,[1] Hanjun Li,[2] Xuyang Zhang,[2] Menghao Yang,[4,*] Ping He,[2,*] Shixue Dou,[1] Yu Ding[2,*]

[1]Institute of Energy Materials Science, University of Shanghai for Science and Technology, Shanghai 200093, China;

[2]National Laboratory of Solid State Microstructures, Collaborative Innovation Center of Advanced Microstructures, College of Engineering and Applied Sciences, Jiangsu Key Laboratory of Nano Technology, Center of Energy Storage Materials & Technology, Nanjing University, Nanjing 210023, China;

[3]Shanghai Key Laboratory for R&D and Application of Metallic Functional Materials, Institute of New Energy for Vehicles, School of Materials Science and Engineering, Tongji University, Shanghai 201804, China;

[4]College of Smart Energy, Shanghai Jiao Tong University, Shanghai 200240, China

***Corresponding authors.** E-mails: yuding@nju.edu.cn; pinghe@nju.edu.cn; menghaoyoung@tongji.edu.cn

[†]Equally contributed to this work.

Supplementary Information includes:

Supplementary Figures 1 to 22

Supplementary Tables 1 to 2

Methods

Regeneration of cathode materials (NCM, LCO, LFP, and LMO)

The regeneration of NCM, LCO, LFP, and LMO cathode materials was performed through a two-step process involving pretreatment and calcination. For NCM, LCO, and LMO, the separated materials underwent an initial sintering process at 600 °C for 4 hours in a muffle furnace. Subsequently, 10 wt.% lithium carbonate (Li_2CO_3) was added to the materials, and the mixture was homogenized. The restoration was then completed by calcination at 800 °C for 12 hours for NCM, 900 °C for 9 hours for LCO, and 850 °C for 6 hours for LMO. For LFP cathode materials, the materials were mixed with 5 wt.% glucose and 10 wt.% Li_2CO_3, followed by calcination under an argon atmosphere at 700 °C for 6 hours to complete the restoration process. Glucose serves two functions. First, it provides an in-situ carbon source to form a conductive carbon coating on regenerated $LiFePO_4$, which is essential given the intrinsically low electronic conductivity of olivine phosphates. Second, the decomposition of glucose generates a mildly reductive local atmosphere, which suppresses the oxidation of Fe^{2+} to Fe^{3+} and prevents the formation of impurity phases.

Characterization of the GaSn-Al Composites

The morphology and composition of the composite were characterized using a Hitachi S-4800 scanning electron microscope (SEM). X-ray diffraction (XRD) analysis was conducted on a D8 Advance with Cu Kα radiation. Diffraction data were collected over a 2θ range of 10° to 90° with a scan rate of 3 min^{-1}. Differential scanning calorimetry (DSC) apparatus (NETZSCH Q20, Thermal Analysis, USA) was used to characterize the melting and crystallization properties of GaSn-Al alloy.

Determination of GaSn and Al Concentrations

The concentrations of GaSn and Al were determined using inductively coupled plasma optical emission spectroscopy (ICP-OES) on Nu Plasma 1700 ICP spectrometer, with scandium and yttrium employed as internal standards. Calibration curves for Ga, Sn, and Al were generated using external standard solutions.

Viscosity measurement

The viscosity of GaSn liquid alloy with different Al contents was measured using a rotational viscometer equipped with an IV rotor. The measurements were conducted at a rotational speed of 60 rpm, and viscosity curves were obtained for each sample over the temperature range of 20–70 °C.

Life cycle assessment (LCA) analysis

The EverBatt model developed by the Argonne National Laboratory was used for the LCA and techno-economic analysis (TEA) of the above recycling processes based on the treatment of 10,000 tons of spent NCM333 cells.

Density functional theory (DFT) calculation

All the structural optimizations and energy calculations were carried out based on the DFT as implemented in Vienna Ab initio Simulation Package (VASP)[1]. The projector-augmented wave (PAW)[2] method was implemented to calculate the interaction between the ionic cores and valence electrons. Perdew-Burke-Ernzerhof (PBE)[3] approach of spin-polarized generalized gradient approximation was used to describe the exchange-correlation energy. The structure of Ga_9Sn was generated based on the structure of Ga_3Sn. The structures of Ga_3Sn and Al were got from Materials Project (MP) database[4,5].

Calculations of surface Energy were performed for the heterostructure of Al and Ga_9Sn. The cutoff plane-wave kinetic energy of 520 eV was used in calculations. The conjugate-gradient method was used in electronic relaxation, with the total energy convergence criterion is 10^{-2} eV. Gamma-point-centered k-meshes were used for all calculations, and the *k*-point grid size was [1 1 1]. The formula for calculating binding energy is as follows:

$$E_{\mathrm{binding}}=(E_{\mathrm{tot}}-E_{\mathrm{Al}}-E_{(\mathrm{Ga_9Sn})})/2 \quad (1)$$

where E_{surface} is surface energy. E_{tot} is the energy of heterostructure of Al and Ga_9Sn. E_{Al} is the energy of Al and $E_{(\mathrm{Ga_9Sn})}$ is the energy of Ga_9Sn.

The Al-doped Ga_9Sn system is modeled based on the Ga_9Sn structure. Ga and Sn atoms, in a 9:1 ratio, are randomly selected and replaced by an equal number of Al atoms. For each Al doping concentration, multiple doping configurations were tested, followed by structural optimization. The most stable configuration was selected based on the results of these optimizations.

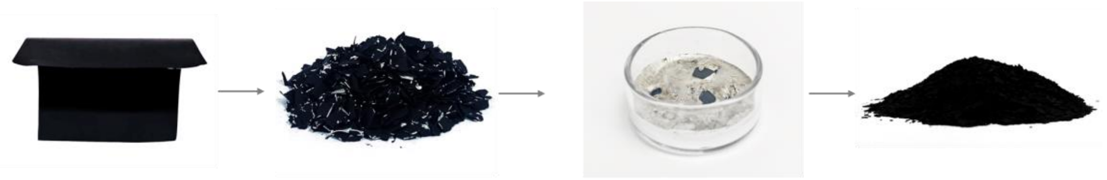

Figure S1. Digital photographs illustrate the liquid metal-enabled electrode separation and regeneration cycle. The process includes: 1) the intact spent electrode sheet; 2) mechanically shredded electrode materials; 3) electrode materials immersion; 4) separated electrode materials.

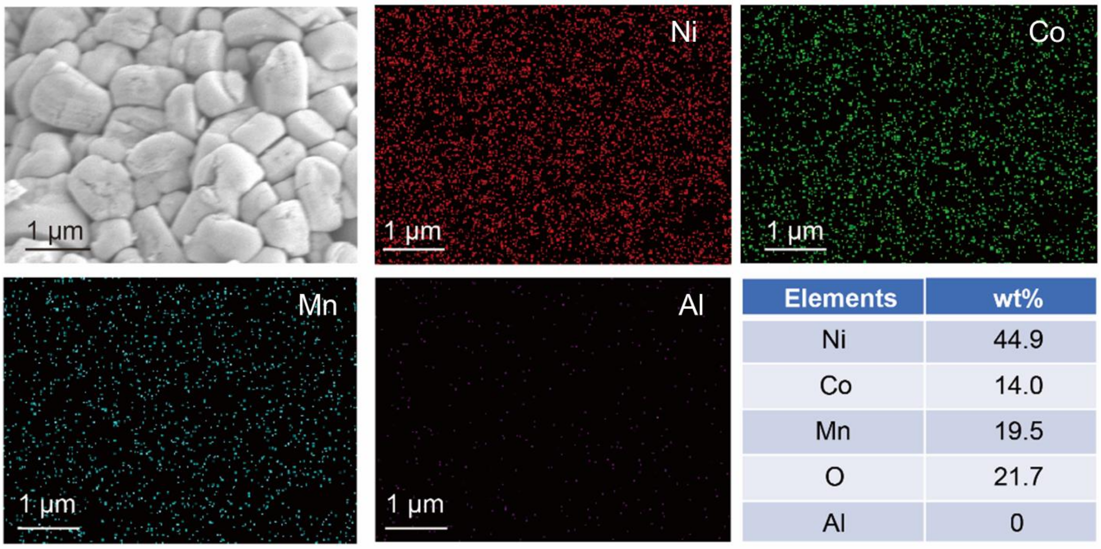

Elements	wt%
Ni	44.9
Co	14.0
Mn	19.5
O	21.7
Al	0

Figure S2. EDS analysis of the separated active materials, showing elemental mapping and weight percentages of each element (Ni, Co, Mn, O, and Al).

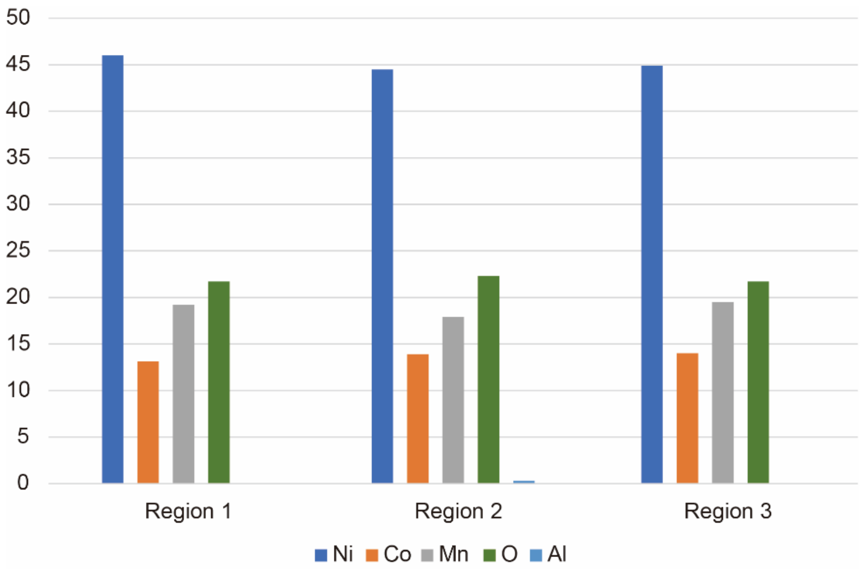

Figure S3. EDS analysis of different regions on the separated active materials, showing negligible presence of Al.

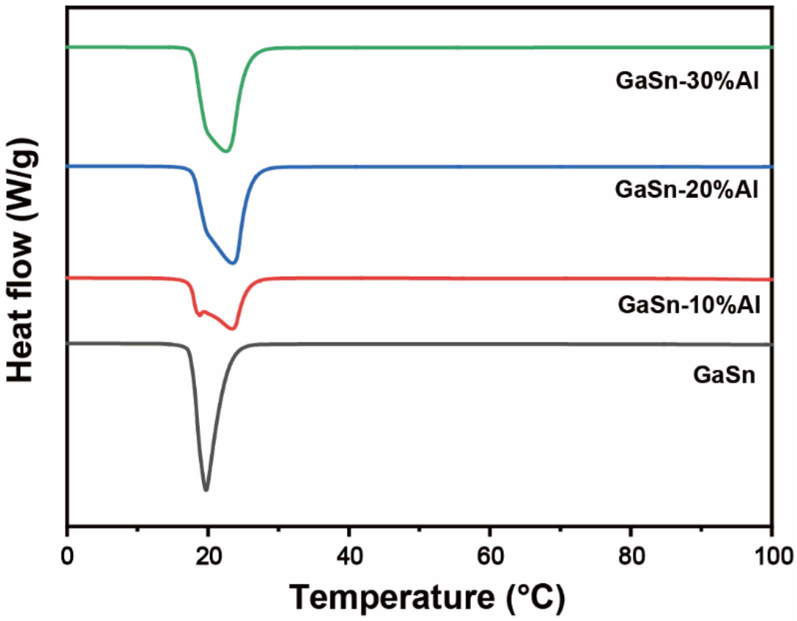

Figure S4. Differential scanning calorimetry characterization of the GaSn-Al system, showing its low melting point and confirming that the system remains in a liquid state at room temperature despite the increased viscosity.

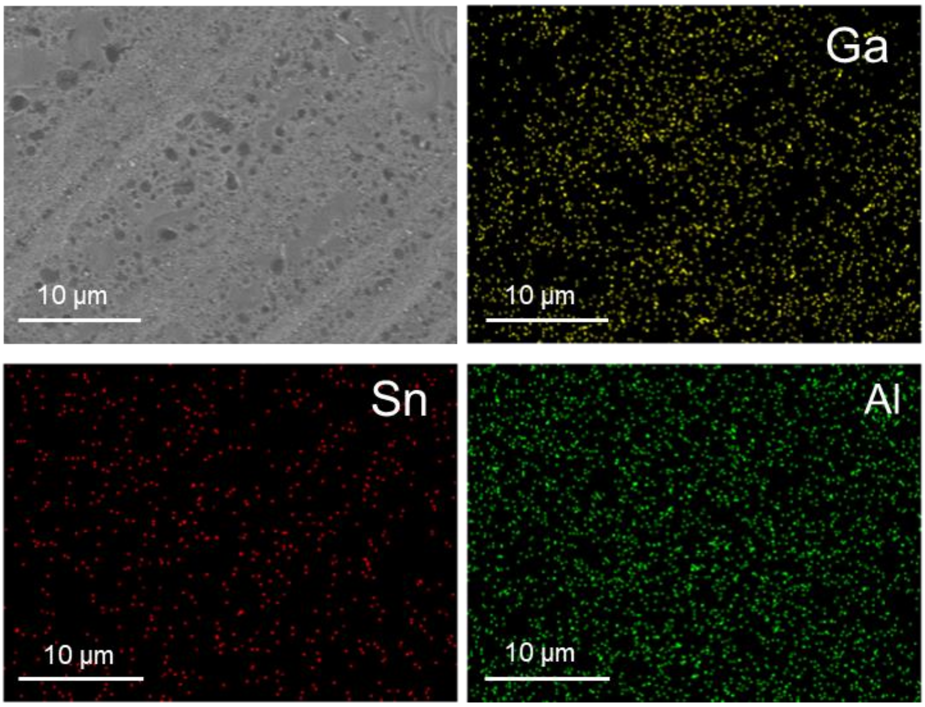

Figure S5. SEM characterization of 10 at.% Al in GaSn alloy. The SEM image shows the uniform distribution of Al in the GaSn matrix, with no observable segregation, indicating a homogeneous alloy composition at the microstructural level.

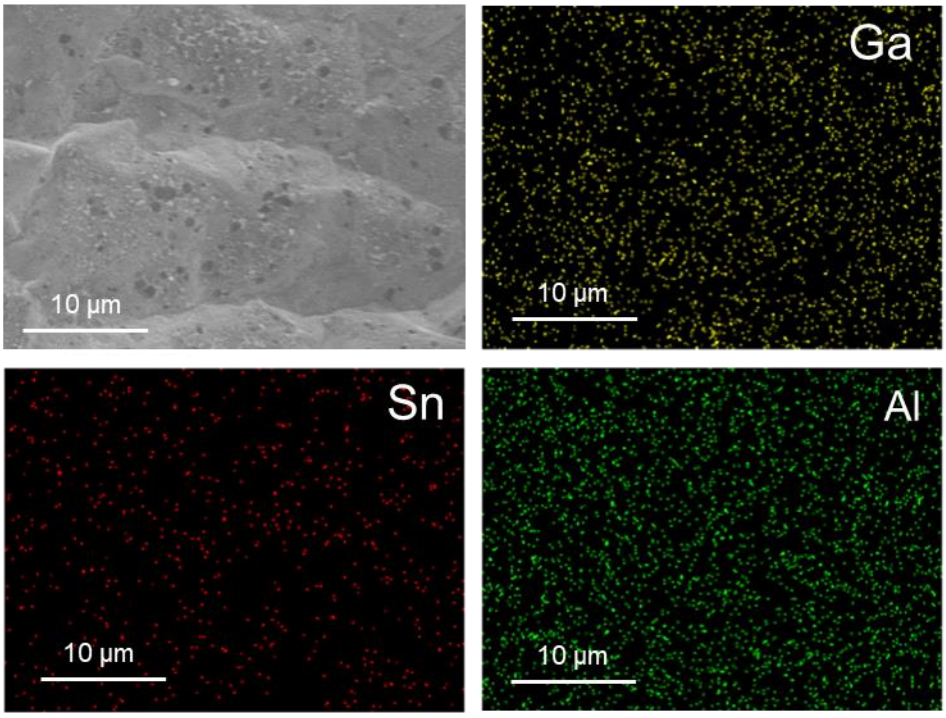

Figure S6. SEM characterization of 20 at.% Al in GaSn alloy. The SEM image also shows the uniform distribution of Al in the GaSn matrix, with no signs of segregation.

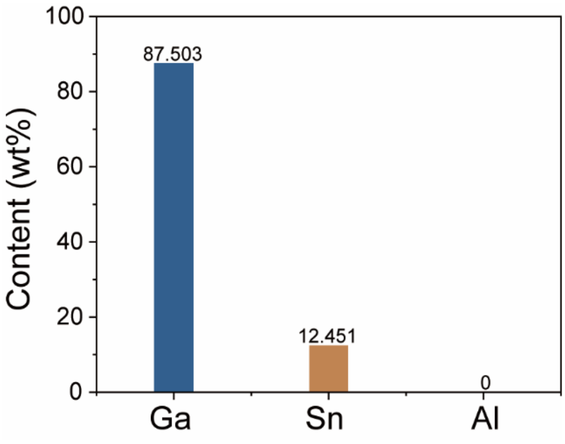

Figure S7. ICP-MS analysis of the original GaSn alloy.

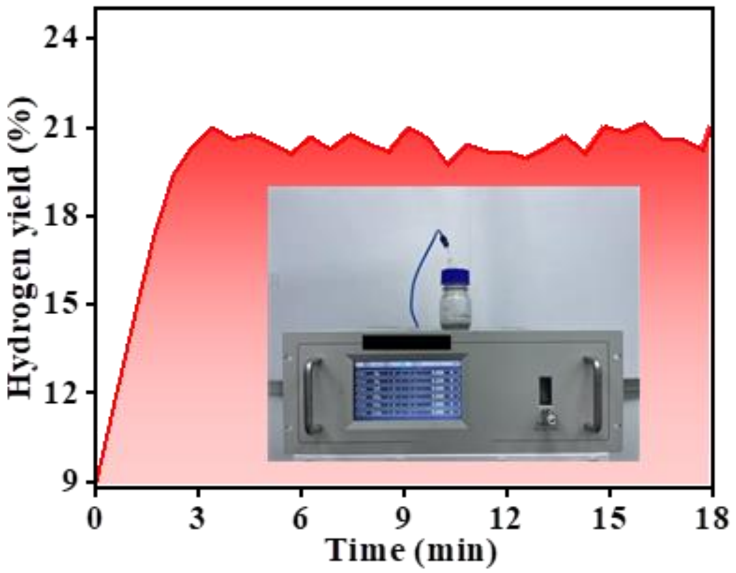

Figure S8. Digital photo of the gas product testing equipment during data collection. The image shows the setup used for monitoring gas production. Time-dependent hydrogen production curve, showing a continuous and stable hydrogen gas evolution over time, indicating sustained gas output throughout the testing period.

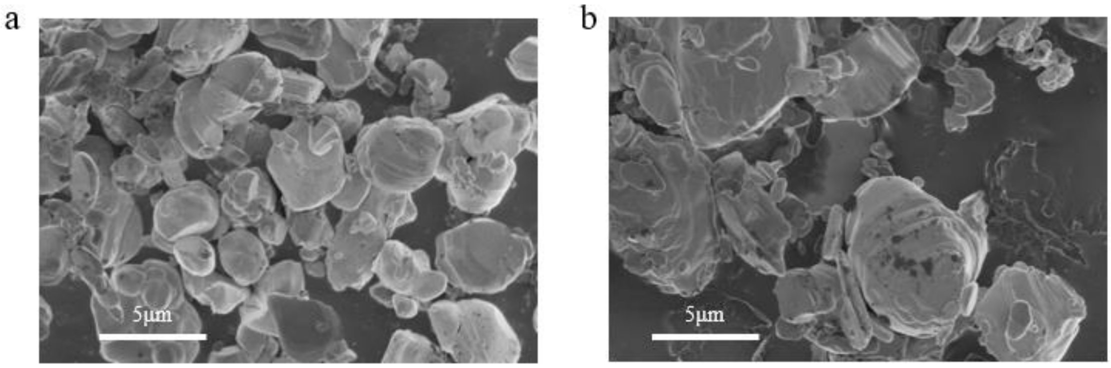

Figure S9. SEM images of spent (a) and regenerated LCO (b).

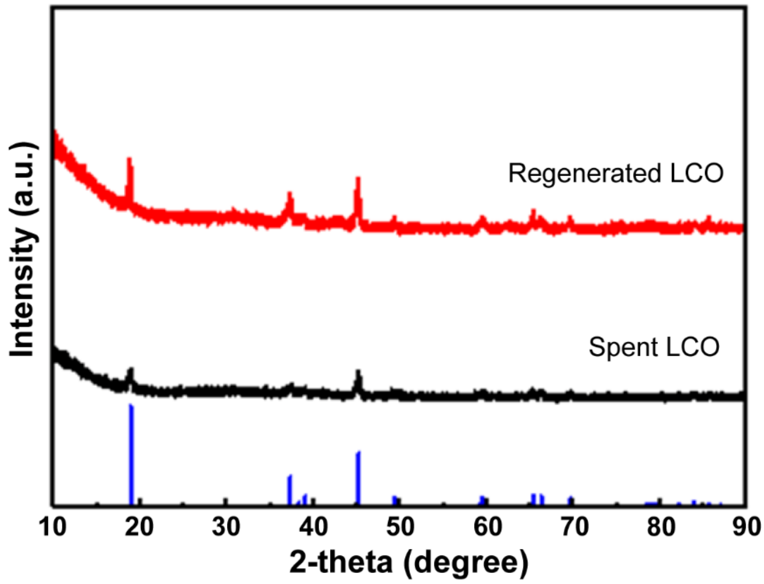

Figure S10. XRD patterns of the spent LCO (black) and regenerated LCO (red).

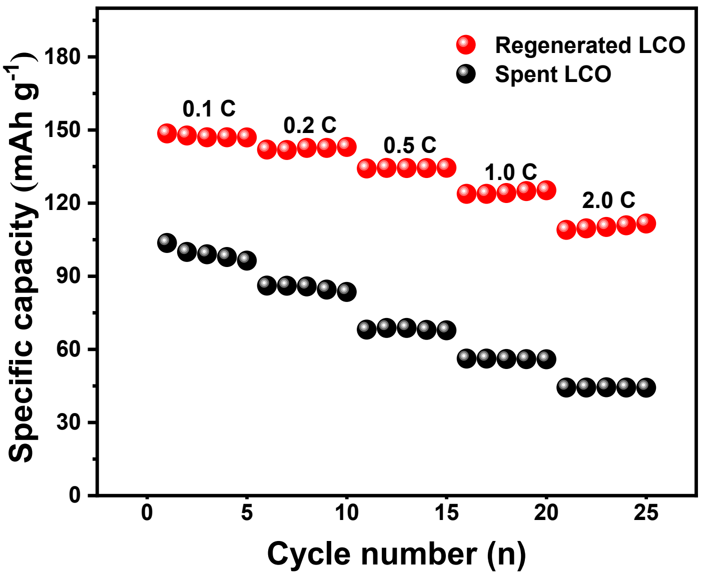

Figure S11. Rate performance at various current densities (0.1, 0.2, 0.5, 1.0, and 2.0 *C*) for the spent and regenerated LCO.

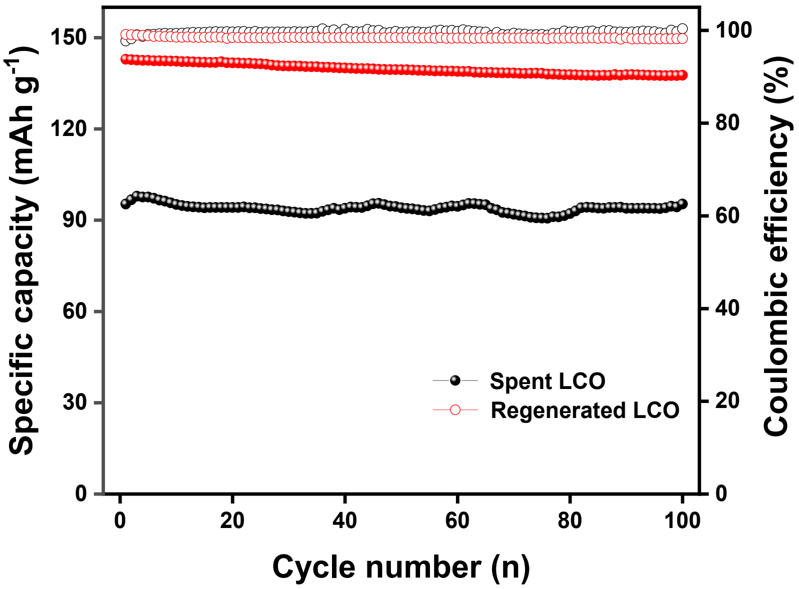

Figure S12. Cycling performance at 0.2 *C* for the spent and regenerated LCO.

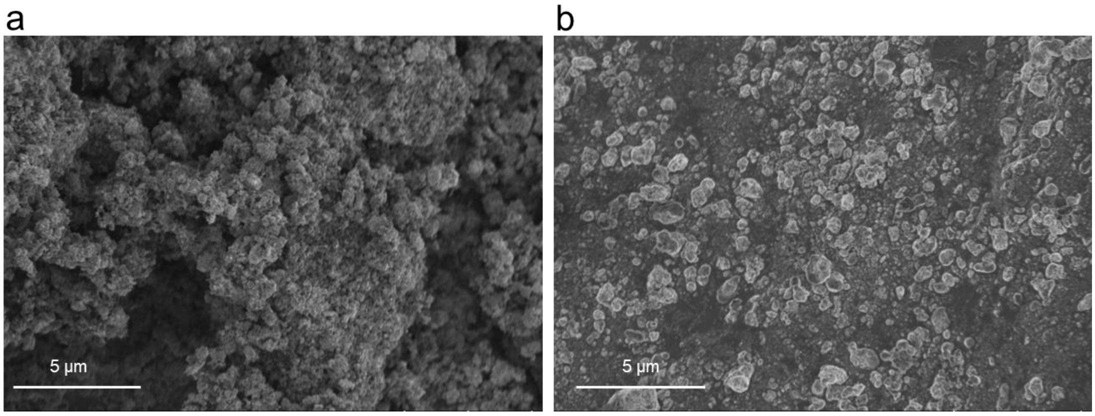

Figure S13. SEM images for the spent (a) and regenerated LFP (b).

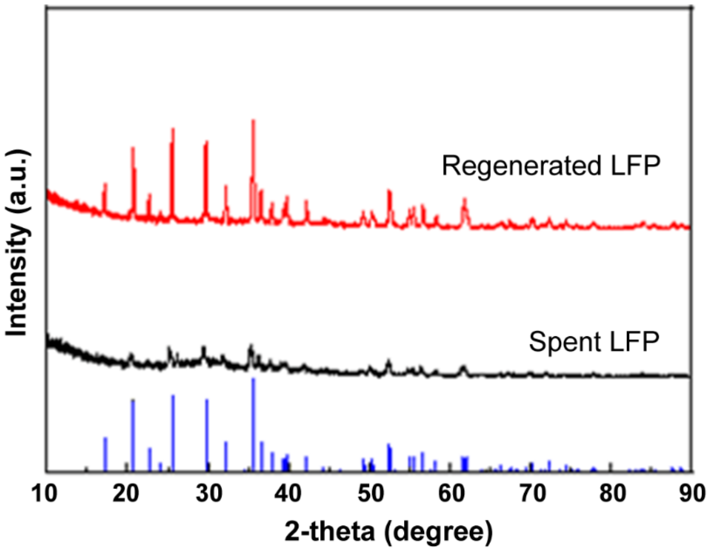

Figure S14. XRD patterns of the spent LFP (black) and regenerated LFP (red).

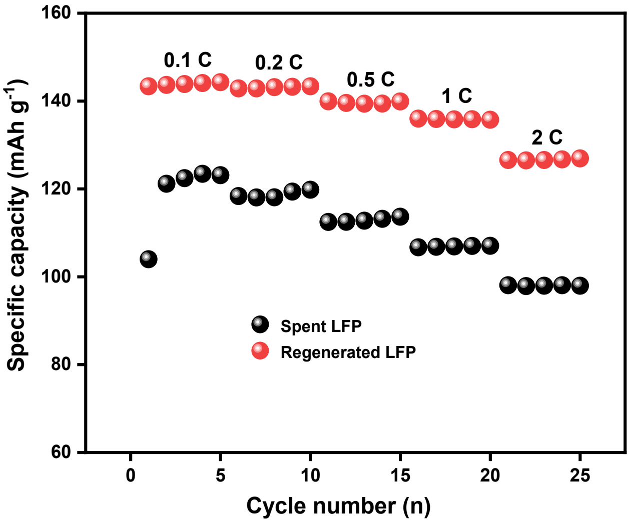

Figure S15. Rate performance at various current densities (0.1, 0.2, 0.5, 1.0, and 2.0 *C*) for the spent and regenerated LFP.

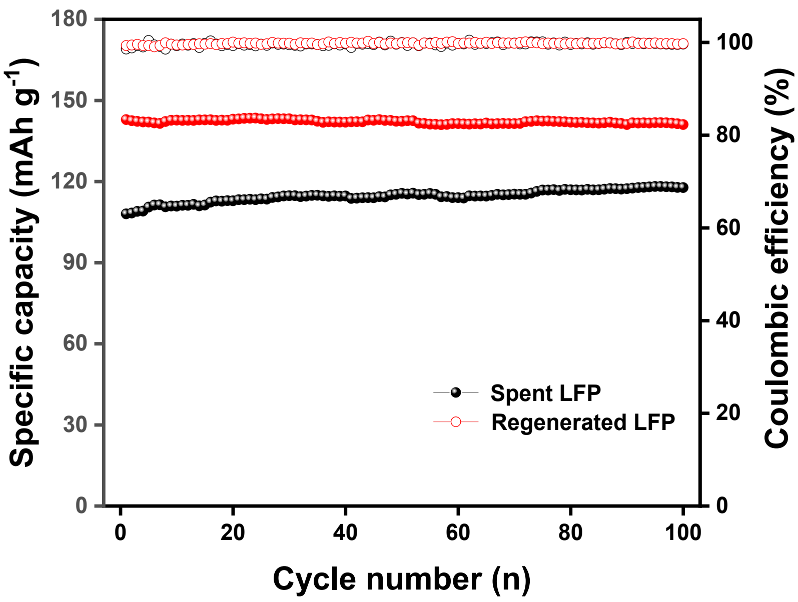

Figure S16. Cycling performance at 0.2 *C* for the spent and regenerated LFP.

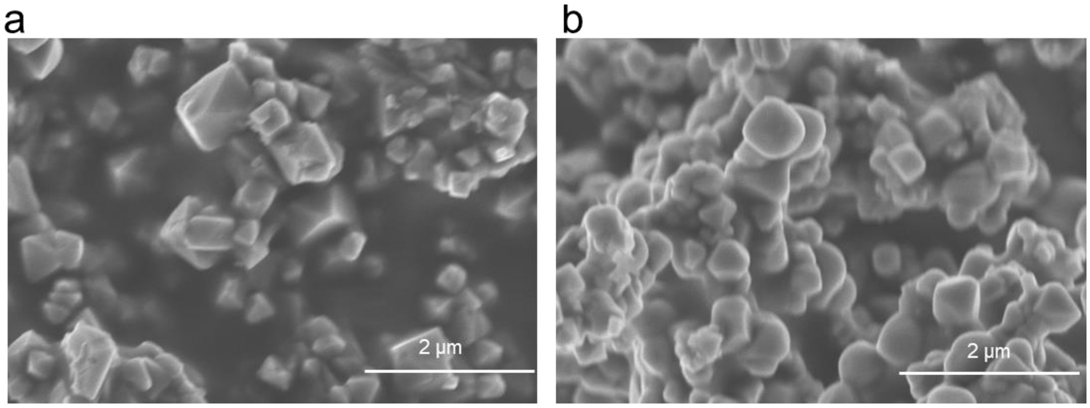

Figure S17. SEM images for the spent (a) and regenerated LMO (b).

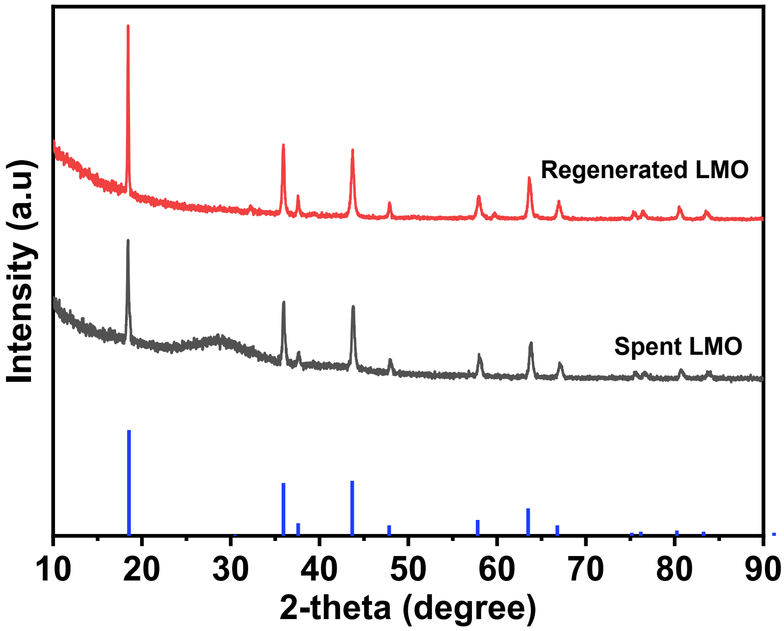

Figure S18. XRD patterns of the spent (black) and regenerated LMO (red).

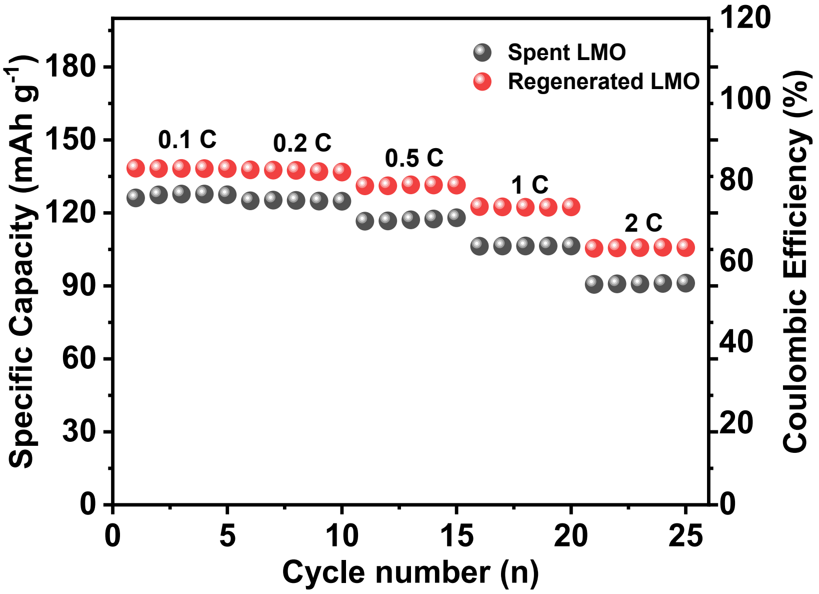

Figure S19. Rate performance at various current densities (0.1, 0.2, 0.5, 1.0, and 2.0 *C*) for the spent and regenerated LMO.

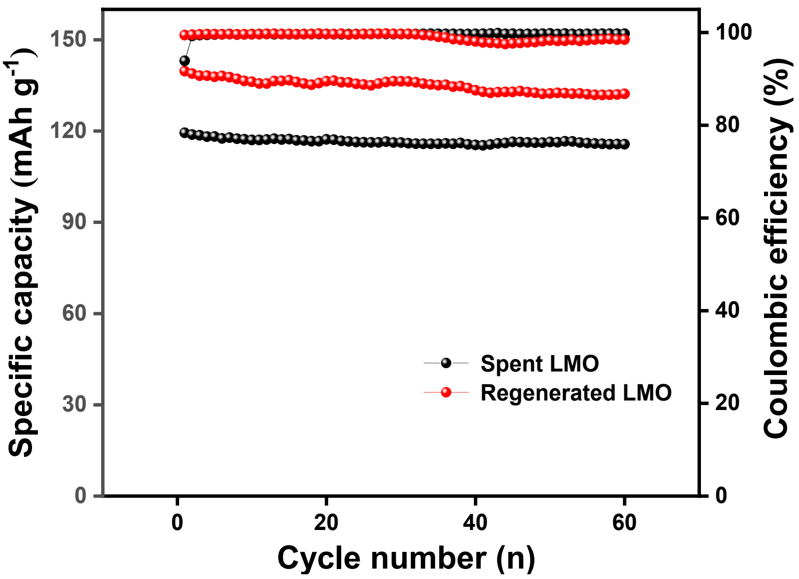

Figure S20. Cycling performance at 0.2 *C* for the spent and regenerated LMO.

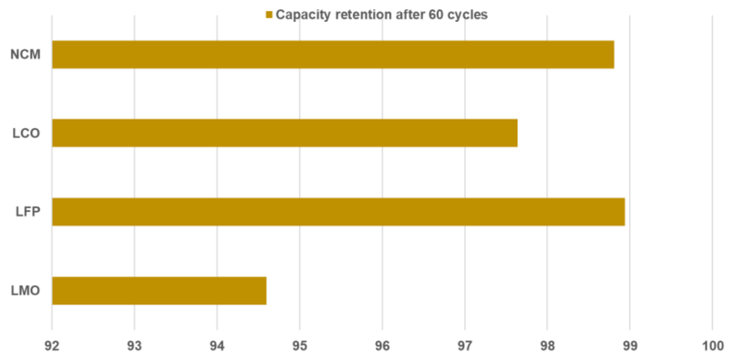

Figure S21. Capacity retention after 60 cycles of regenerated cells for NCM, LCO, LFP, and LMO.

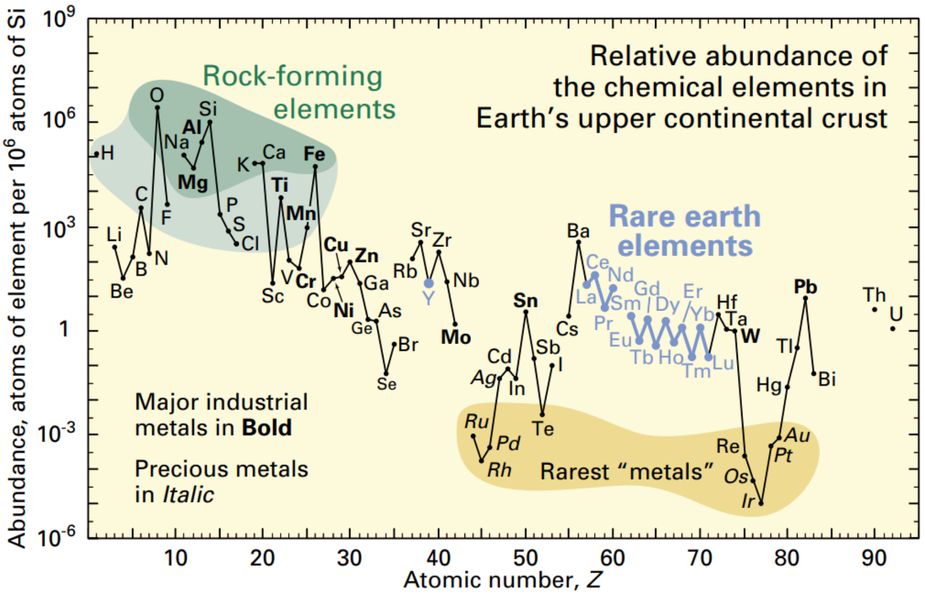

Figure S22. Abundance of the chemical elements in Earth's crust as a function of atomic number. (G. B. Haxel, J. B. Hedrick, G. Orris, Rare Earth Elements Critical Resources for High Technology, 2002.)

References

1. Kresse G, Furthmüller J, Efficient iterative schemes for ab initio total-energy calculations using a plane-wave basis set. *Phys. Rev. B* 1996, **54**, 11169.
2. Blöchl PE, Projector augmented-wave method. *Phys. Rev. B* 1994, **50**, 17953.
3. Perdew JP, Ernzerhof M, Burke K, Rationale for mixing exact exchange with density functional approximations. *J. Chem. Phys.* 1996, **105**, 9982.
4. Jain A, Hautier G, Moore CJ, Ong SP, Fischer CC, T. Mueller, K. A. Persson, G. Ceder, A high-throughput infrastructure for density functional theory calculations. *Comput. Mater. Sci*. 2011, **50**, 2295.
5. Jain A, Ong SP, Hautier G, Chen W, Richards WD, Dacek S, Cholia S, Gunter D, Skinner D, Ceder G, Persson KA, Commentary: The Materials Project: A materials genome approach to accelerating materials innovation. *APL Mater.* 2013; **1**, 011002.

Table S1. Comparison of representative green solvent, ionic liquid, and DES-based cathode delamination methods.

Category	Representative recent studies	Delamination mechanism	Efficiency and operating conditions	Selectivity and material integrity	Recyclability and environmental impact	Scalability and economic considerations
**Green solvent-based delamination**	Bücken *et al.*, *RSC Adv.*, 2021, 11, 27356; Li *et al.*, *Sep. Purif. Technol.*, 2024, 338, 126625	Dissolution or strong swelling of PVDF binder in green organic solvents	High delamination efficiency reported, but typically requires large solvent volumes and extended soaking times	Limited selectivity: binder dissolution leads to co-dispersion of carbon black and fine particles; downstream solid-liquid separation required	Solvents can be recyclable, but recovery and purification are energy-intensive; solvent loss and wastewater generation remain concerns	Solvent handling, recovery units, and safety management increase process complexity and cost at scale
**Ionic liquid (IL)-based methods**	Hu *et al.*, *Energy Environ. Sci.*, 2024, 4238	Ionic liquid (imidazolium glycol) induces delithiation and lattice-oxygen redox, reducing Co(III) → Co(II) and dissolving cathode oxides	Long leaching duration (up to 20-24 h) to reach >99 % metal leaching; typically 120 °C	Poor intrinsic selectivity; Al foil remains as residue but not the focus of separation quality	High cost of ILs; recyclability possible but purity degradation over cycles is an issue	Limited by viscosity, heat transfer, long residence time, and reactor design
**Deep eutectic solvent (DES)-based delamination**	Wang *et al.*, *Energy Environ. Sci.*, 2024, 17, 867; various ChCl-based DES systems	Chemical/thermal degradation or swelling of PVDF and interfacial weakening	Very high efficiencies reported (often >99%), but frequently at high temperatures (e.g., ~180-190 °C)	Moderate selectivity; cathode powder often mixed with binder residues, requiring post-treatment	DESs are often promoted as “green”, but thermal stability, viscosity increase, and solvent regeneration remain challenges	High operating temperature and multi-step solvent recycling complicate scale-up and economics
**Electrochemical / non-solvent reference methods**	Wang *et al.*, *Nat. Sustain.*, 2025, 8, 520 (water electrolysis); Chen *et al.*, *Nat. Commun.*, 2023, 14,	Gas-bubble-induced delamination or interfacial chemical passivation	Rapid delamination with high efficiency (>99%); low energy input reported	High selectivity with minimal damage to active materials and Al foil	Excellent environmental performance (water-based, recyclable electrolytes)	Requires electrochemical infrastructure; process control needed for uniform large-scale treatment

Category	Representative recent studies	Delamination mechanism	Efficiency and operating conditions	Selectivity and material integrity	Recyclability and environmental impact	Scalability and economic considerations
	4648 (reaction-passivation)					
**This work: Liquid-metal-assisted delamination**	*This manuscript*	**Selective interfacial infiltration and wetting of liquid metal at the Al foil-coating interface, enabling mechanical/interfacial separation without dissolving the binder**	**High delamination efficiency achieved under comparatively mild conditions (temperature/time to be specified in this work)**	**Intrinsic selectivity: separation occurs at the current-collector interface rather than through binder dissolution, preserving cathode crystal structure and Al foil integrity**	**Liquid metal is non-volatile, physically recoverable, and reusable over multiple cycles (demonstrated in this work), with minimal solvent waste**	**Simple process flow (no solvent dissolution/recovery steps), favorable for continuous processing and large-scale implementation, with reduced operational and environmental costs**

Note: PVDF = poly(vinylidene difluoride) binder; ChCl = choline chloride

Table S2. Qualitative comparison of key process attributes and technical barriers between conventional recycling methods and the liquid metal-enabled separation strategy.

	**Pyrometallurgy**	**Hydrometallurgy**	**Direct Recycling**	**Liquid Metal Approach**
**Separation Mechanism**	Thermal reduction (smelting entire modules; organic components are burned off)	Chemical leaching (dissolution of active materials using strong acids/bases)	Binder dissolution (chemical solvation of PVDF binder to detach coating)	Intergranular embrittlement (physical disruption of Al grain boundaries and interface)
**Operating Conditions**	>1400 °C	room temp to 80 °C	80-180°C	Ambient/room temperature
**Al Current Collector**	Lost/slag: oxidized into slag; difficult to recover; energy loss	Dissolved/impurity: dissolves consuming acid; requires complex removal steps	Intact: recovered, but often requires washing to remove solvent residues	Embrittled & converted: separated as grains; converted to $Al(OH)_3$ + H_2 (Value-added)
**Cathode Structure**	Destroyed: reduced to alloy (Co, Ni) and slag (Li, Al, Mn)	Destroyed: broken down into ionic solutions (Li^+, etc.)	Preserved: crystal structure maintained; ready for regeneration	Preserved: crystal structure maintained; ready for regeneration
**Environmental Impact**	High GHG emissions: toxic gas release (HF, CO); high energy consumption	High water/acid usage: large volumes of toxic wastewater; secondary pollution risk	VOC emissions: Use of toxic/expensive organic solvents; solvent recovery is energy-intensive	Eco-friendly: solvent-free; negligible GHG; closed-loop liquid metal use; H_2 byproduct
**Processing Speed (Kinetics)**	Slow: long heating and cooling cycles	Medium: leaching takes hours; multiple precipitation steps	Slow: binder dissolution is diffusion-limited	Extremely fast: reaction propagates in <30 mins due to rapid wetting.
**Key Technical/Economic Barriers**	High energy cost; low recovery rate of Li/Al; gas treatment cost.	Complex wastewater treatment; high reagent cost; low selectivity.	Solvent cost and toxicity; binder removal efficiency; scale-up of solvent recovery	Initial material cost of Ga (mitigated by high recyclability); new process integration
